# Development of Silicalite/Glucose Oxidase-Based Biosensor and Its Application for Glucose Determination in Juices and Nectars

**DOI:** 10.1186/s11671-016-1275-2

**Published:** 2016-02-03

**Authors:** Oleksandr Ye Dudchenko, Viktoriya M. Pyeshkova, Oleksandr O. Soldatkin, Burcu Akata, Berna O Kasap, Alexey P. Soldatkin, Sergei V. Dzyadevych

**Affiliations:** Laboratory of Biomolecular Electronics, Institute of Molecular Biology and Genetics, National Academy of Sciences of Ukraine, 150 Zabolotnogo St., 03143 Kyiv, Ukraine; Institute of High Technologies, Taras Shevchenko National University of Kyiv, Volodymyrska Street 64, 01601 Kyiv, Ukraine; Micro and Nanotechnology Department, Central Laboratory, Middle East Technical University, 06530 Ankara, Turkey

**Keywords:** Enzyme immobilization, Silicalite, Glucose oxidase, Conductometric transducer, Biosensor

## Abstract

The application of silicalite for improvement of enzyme adsorption on new stainless steel electrodes is reported. Glucose oxidase (GOx) was immobilized by two methods: cross-linking by glutaraldehyde (GOx-GA) and cross-linking by glutaraldehyde along with GOx adsorption on silicalite-modified electrode (SME) (GOx-SME-GA). The GOx-SME-GA biosensors were characterized by a four- to fivefold higher sensitivity than GOx-GA biosensor. It was concluded that silicalite together with GA sufficiently enhances enzyme adhesion on stainless steel electrodes. The developed GOx-SME-GA biosensors were characterized by good reproducibility of biosensor preparation (relative standard deviation (RSD)—18 %), improved signal reproducibility (RSD of glucose determination was 7 %), and good storage stability (29 % loss of activity after 18-day storage). A series of fruit juices and nectars was analyzed using GOx-SME-GA biosensor for determination of glucose concentration. The obtained results showed good correlation with the data of high-performance liquid chromatography (HPLC) (*R* = 0.99).

## Background

Today, a number of techniques of enzyme immobilization is developed and well-explored in biomedicine, environmental science/engineering, microbial synthesis, and, especially, biosensors. Physical adsorption on a certain carrier is one of the oldest and simplest methods. Usually, enzyme adsorption implies neither additional chemical reagents nor activators; therefore, this is the least denaturing method of immobilization, which provides retention of the enzyme activity. Additionally, adsorption is commercially attractive due to its low cost if compared with other immobilization methods. Lately, use of different nanostructured materials became one of the most common approaches in immobilization techniques [1]. Different zeolites were found to be suitable for this aim, due to their properties [2–4].

Zeolites are hydrated microporous crystalline aluminosilicates. They are composed mainly of silicon, aluminum, and oxygen. Modification of crystal structures makes it possible to obtain zeolites with different properties. Silicalites belong to zeolites but differ from other zeolites in the absence of aluminum atoms in their structure. Silicalites were found to be good adsorbents due to the presence of numerous channels in their crystals (and thus silicalites have a large surface area). The presence of hydroxyl groups as well as hydrophilic and hydrophobic sites on the silicalite surface enables numerous interactions between enzyme and particle [5]. Zeolite micropores form a vast and regular network of channels and cages with well-defined size and shape. Furthermore, zeolites are able to exchange ions with some compounds [6].

Nanostructured zeolites are suitable for enzyme immobilization and the engineering of enzyme-based catalysts and biosensors. The micro devices with highly sensitive bio-catalytic functions were designed using enzymes adsorption on nanozeolites owing to their large external surface [4].

For example, it described the improvement of analytical characteristics of the urease-based potentiometric biosensor as a result of using different modifications of Na^+^-Beta and LTA zeolites [7]. It was shown that using of BEA-zeolites increased sensitivity to urea. In [8], the urea and butyrylcholine biosensors were prepared using adsorption of urease and butyrylcholinesterase on heat-treated zeolite beta crystals incorporated in the membranes, which were deposited on the ion-selective field-effect transistor (ISFET) surfaces.

Zeolite beta and silicalite were also applied for preparation of the urease-based conductometric biosensor. Zeolites were added to the immobilization mixture to improve the standard procedure of cross-linking in glutaraldehyde vapor; the urease adsorption without glutaraldehyde was also described. It was demonstrated that use of zeolite allows enhancement of sensitivity and stability of the biosensors constructed [9]. The silicalite-based enzyme biosensor for urea determination in the blood serum reported in [10] showed good correlation with the results of the traditional method of urea measurement based on the diacetyl monoxime reaction (correlation coefficient 0.995).

In [11], the characteristics of the conductometric urea biosensors based on urease adsorbed on silicalite were shown to be better than those of the biosensors based on urease immobilized in glutaraldehyde vapor. Notably, the method of urease adsorption on silicalite is simple and rapid; it does not involve any toxic reagents.

Stainless steel electrodes are quite promising for the development of conductometric biosensors. However, there is a problem of poor enzyme adhesion to the surface of electrodes of this type. To overcome this problem, the electrodes were modified with silicalite, which has good adsorption properties, hydrophobic and organophilic selectivity, and high thermal and chemical stability [12]. The efficiency of enzyme adsorption on such electrodes was evaluated. The biosensor developed was studied under measurement of glucose concentration in real samples of beverages.

## Methods

### Materials

Enzyme glucose oxidase (GOx) from *Penicillium vitale* (ЕС 1.1.3.4) with activity of 130 U/mg was from Diagnosticum (L’viv, Ukraine). Bovine serum albumin (BSA) (V fraction) and 50 % aqueous solution of glutaraldehyde (GA) were obtained from Sigma-Aldrich Chemie (Germany). All the reagents used were of analytical grade and used without further purification.

### Conductometric Transducers

**Fig. 1:**
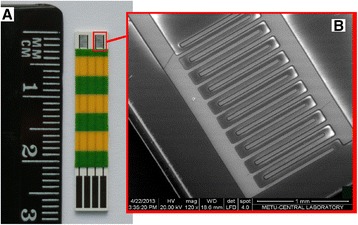
Full view of bare conductometric transducer **(a)**. Scanning electron microscope image of one pair of electrodes **(b)**

Stainless steel transducers were 5 mm × 29 mm in size and consisted of two identical pairs of stainless steel interdigitated electrodes deposited onto a ceramic support (Fig. 1a). The electrodes, consisting of stainless steel and titanium (adhesion) sublayer, were deposited onto the support by thermo vacuum sputtering. The dimensions of the sensitive area of each electrode pair were about 1.5 mm × 2 mm. Each digit and inter-digital space was of 50 μm in width. The scanning electron microscope (SEM) image of the active region of stainless steel transducer is shown in Fig. 1b.

### Synthesis of Silicalite

**Fig. 2:**
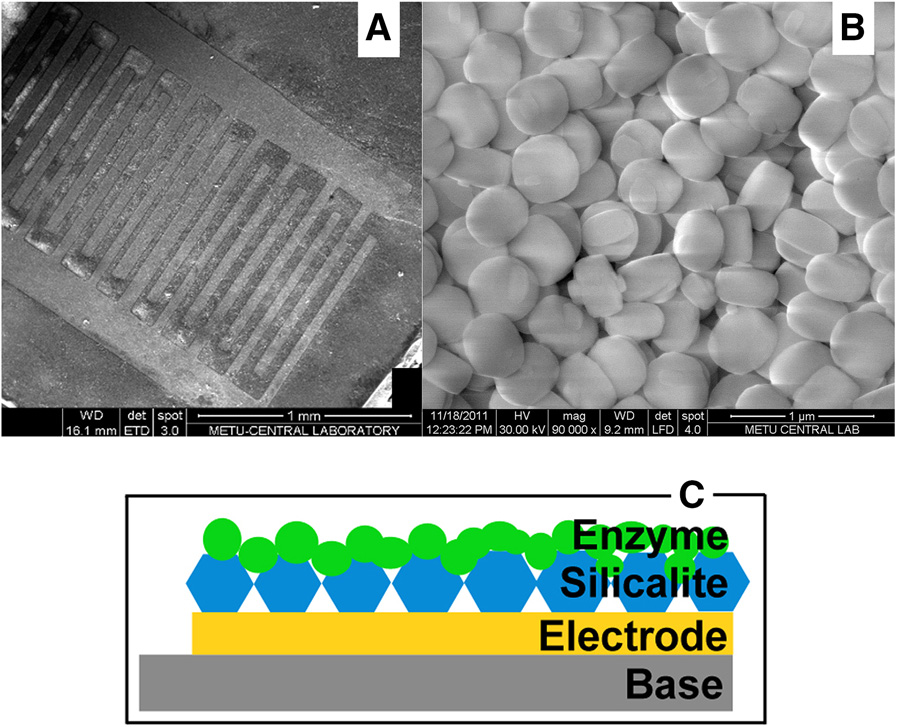
Scanning electron microscope image of conductometric stainless steel electrodes modified with silicalite **(a)** and silicalite particles **(b)**. Schematic side view of prepared biosensor (GOx-SME-GA) **(c)**

Silicalite was synthesized in the Middle-East Technical University (Ankara, Turkey). The optimized molar composition of the gel used for synthesis of silicalite is 1 tetrapropylammonium hydroxide (TPAOH):4 tetraethyl orthosilicate (TEOS):350 H_2_O. TEOS (95 %) was used as the silica source. TPAOH (25 %) was used as a template. A clear homogeneous solution was obtained by hydrolyzing TEOS with TPAOH solution at room 22 °С under 6 h of stirring. Afterwards, the resulting gel was kept in oven for 18 h at 125 °C. To remove the unreacted material, the gel was centrifuged at 13,000 rpm, then washed with deionized water and dried at 80 °C. The SEM image of synthesized silicalite (Fig. 2b) shows that the prepared silicalite particles were of about 400 nm.

### Preparation of Silicalite-Modified Electrode

A silicalite layer on the transducer surface was formed by drop-coating technique. We used 10 % (*w*/*w*) silicalite suspension in 5 mM phosphate buffer, pH 7.0. Constant amount (0.165 ml) of silicalite suspension was deposited in the active zone of each pair of electrodes, and then the transducer was heated for 2 min to 200 °C. This temperature had no effect on the transducer working parameters. The procedure resulted in the formation of silicalite layer in the electrodes active zones (Fig. 2a).

### Bioselective Membrane Preparation

To form a bioselective membrane on electrodes, two types of enzyme immobilization were used: (1) GOx cross-linking with GA on silicalite-modified electrodes (GOx-SME-GA) (Fig. 2c) and (2) GOx cross-linking with GA on bare electrodes (GOx-GA).

To prepare the enzyme membrane, the mixture containing 10 % (*w*/*w*) of GOx, 10 % (*w*/*w*) of BSA, and 20 % (*w*/*w*) of glycerol in 20 mM phosphate buffer, pH 7.0, was used. The mixture for reference membrane was prepared in analogous way, except that GOx was replaced with BSA. Thus, the mixture for reference membrane contained 20 % (*w*/*w*) of BSA. Each solution was separately mixed with 1 % (*w*/*w*) aqueous solution of GA in a ratio of 1:1. Immediately afterwards, the mixture of enzyme with GA was deposited on one pair of electrodes and the mixture of reference solution with GA—on another. In 20 min, strong covalent bonds between the compounds of bioselective membrane were formed. After immobilization, the electrodes were submerged in the working buffer for 30 min to wash out the unbound enzyme and GA excess. SME was used as a carrier to obtain GOx-SME-GA biosensor, and a bare electrode (without silicalite)—in GOx-GA biosensor.

### Measurement Procedure

The measurements were carried out in an open electrochemical cell at 21–24 °C with continuous stirring. Five millimolar phosphate solution, pH 6.5, was used as a working buffer solution. The required substrate concentration in the working buffer was achieved by adding an aliquot of the stock substrate solution. The experiments were reproduced at least three times sequentially. The effects of temperature and pH changes and electric interferences were avoided by operating in the differential mode.

### High-Performance Liquid Chromatography

The high-performance liquid chromatography (HPLC) method was performed using Varian ProStar HPLC system (Agilent Technologies, USA) along with ProStar 350 Refractive Index Detector (Agilent Technologies, USA). Additional parameters were max flow rate—20 mL/min and flow cell volume—7 μl. It is a fully integrated and automated system that could be controlled in four different modes. In HPLC, the purity is very important factors for the accuracy of the results. For this reason, the samples were thoroughly cleaned from all impurities and filtered through a suitable filtration system (0.45 μm). Column Microsorb-MV NH_2_ (4.6 × 250 mm) (Agilent Technologies, USA), applicable for saccharides, was used. Eighty percent acetonitrile (*v*/*v*, in water) (Sigma-Aldrich Chemie, Germany) was used as a solvent.

## Results and Discussion

Conductometric glucose biosensors rely on the changes in conductivity associated with the enzymatic oxidation of glucose according to the following formula:

$$ \begin{array}{l}\kern12.22em \mathrm{Glucose}\\ {}\kern12.22em \mathrm{oxidase}\\ {}\beta \hbox{-} \mathrm{D}\hbox{-} \mathrm{Glucose}+{O}_2+{\mathrm{H}}_2O\kern1.32em \to \kern1.2em \mathrm{D}\hbox{-} \mathrm{Glucono}\hbox{-} \delta \hbox{-} \mathrm{lactone}+{\mathrm{H}}_2{O}_2\\ {}\kern17.7em \uparrow \downarrow \\ {}\kern16em \mathrm{D}\hbox{-} \mathrm{Gluconate}+{\mathrm{H}}^{+}\end{array} $$

d-Gluconolactone is spontaneously hydrolyzed to gluconic acid, which dissociates to the acid residue and a proton. These reactions lead to changes in solution conductivity that can be registered by the conductometric system.

**Fig. 3:**
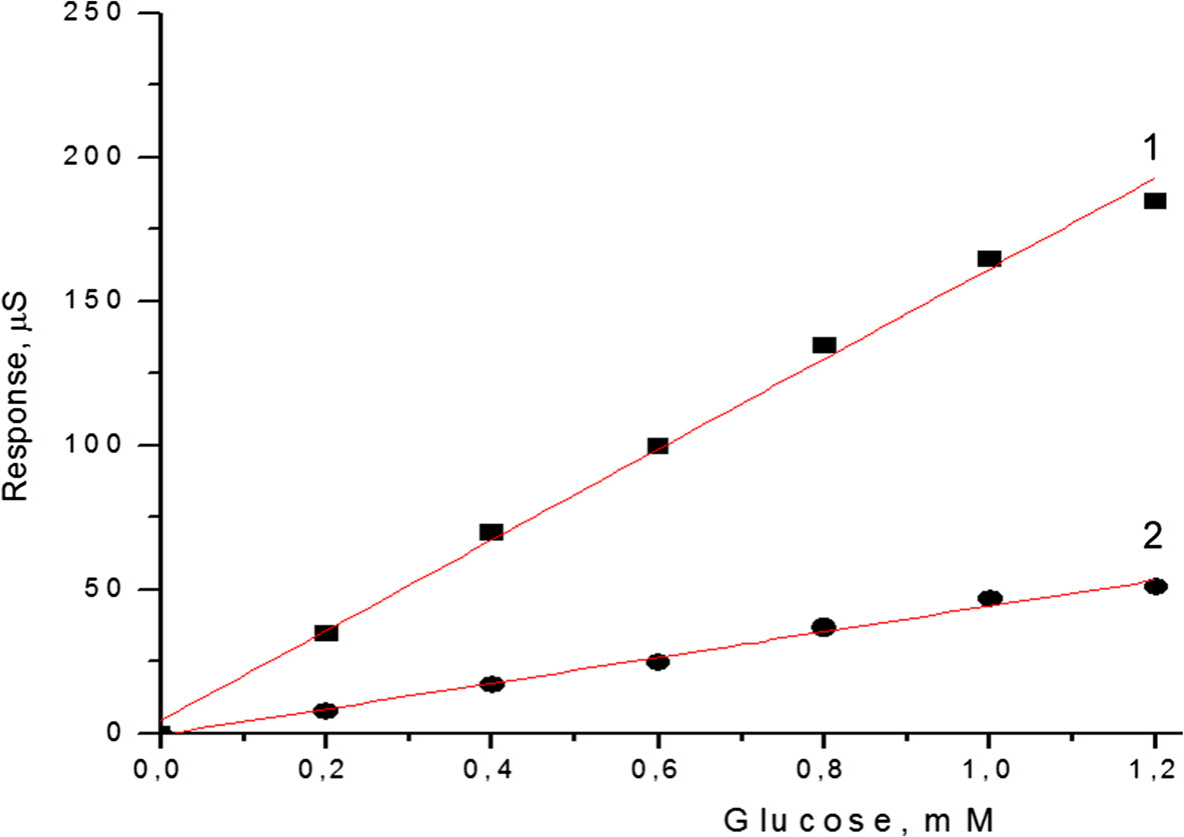
Calibration curves of glucose conductometric biosensors with different types of GOx immobilization: *1* GOx-SME-GA, *2* GOx-GA. Measurements were carried out in 5 mM phosphate buffer, pH 6.5

First, the calibration curves were plotted for the biosensors with both types of immobilization (Fig. 3). As seen, the linear range of glucose measurement for both GOx-SME-GA and GOx-GA biosensors was 0.005–1.2 mM, but the GOx-SME-GA biosensor had 4–5 times higher sensitivity to glucose.

### Reproducibility of Biosensors Preparation and Signal Reproducibility

**Fig. 4:**
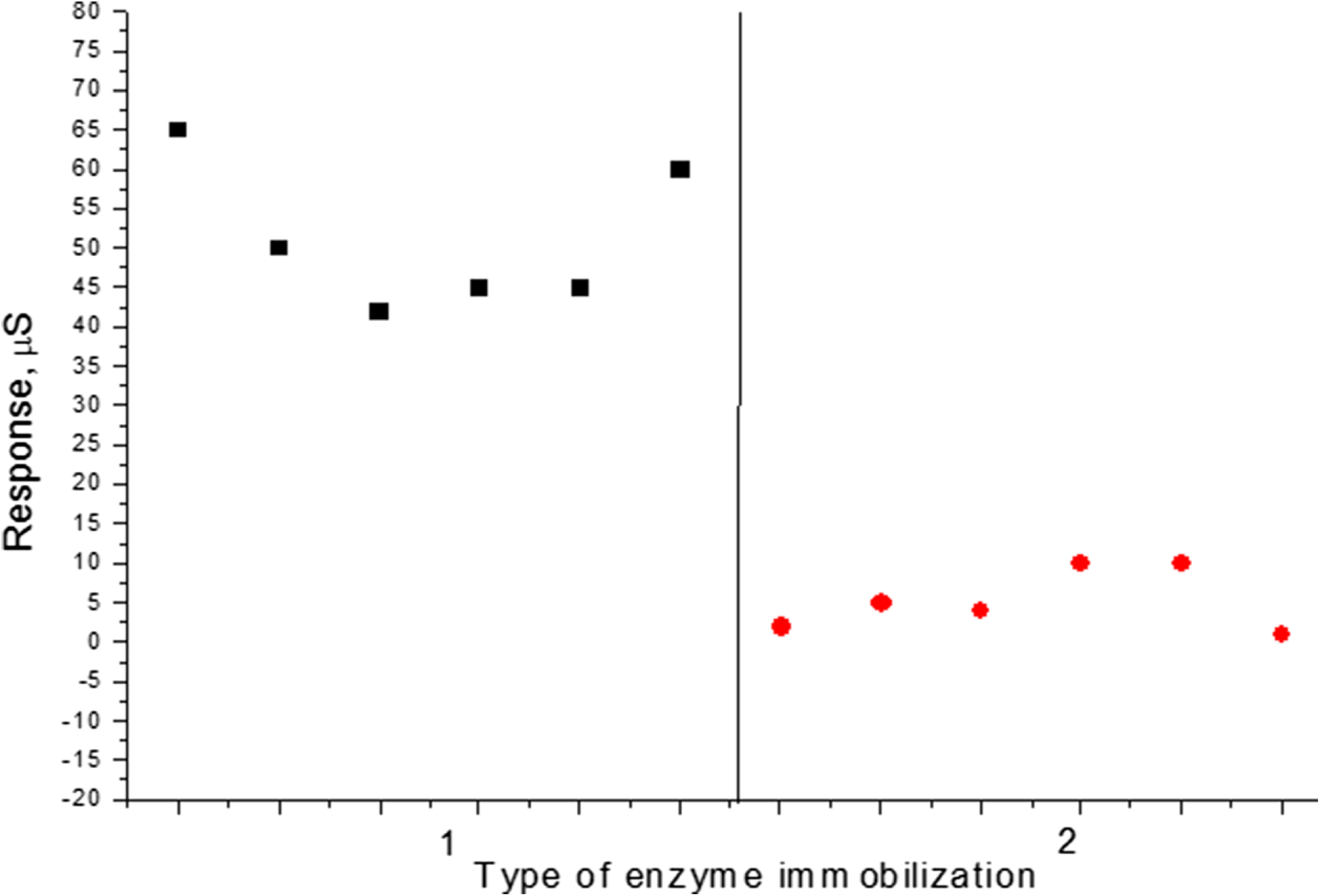
Reproducibility of glucose biosensor preparation based on stainless steel electrodes with different types of GOx immobilization: *1* GOx-SME-GA, *2* GOx-GA. Measurements were carried out in 5 mM phosphate buffer, pH 6.5; glucose concentration was 0.2 mM

Reproducibility of biosensors preparation is an important performance property, which sets conditions for the biosensor standardization. Therefore, this parameter was checked for both groups of biosensors. To investigate reproducibility of biosensors preparation, the group of biosensors was prepared (in amount of six for both GOx-SME-GA and GOx-GA type of biosensors) and then their responses to 0.2 mM glucose were obtained. The error (RSD) of biosensors preparation for the GOx-SME-GA biosensors was found to be 18 %, for GOx-GA biosensors—76 % (Fig. 4), i.e., the technique of preparation of GOx-SME-GA biosensors is more reproducible.

Therefore (and taking into account that earlier they were shown to have a higher sensitivity to glucose), the GOx-SME-GA biosensors were under further investigation.

To study signal reproducibility, the biosensor responses to 0.2 mM glucose were measured over one working day every 10–15 min. During the measurements, the biosensors have been kept in the continuously stirred buffer solution. The error (RSD) of glucose measurements has been found to be 7 % that is quite acceptable.

Notably, reproducibility of biosensors preparation strongly depends on the enzyme immobilization techniques involved, i.e., whether it is an automatic or staff-required process. Thus, it could be a subject of consideration in future when commercialization would be an actual challenge.

### Storage Stability of GOx-SME-GA Biosensors

**Fig. 5:**
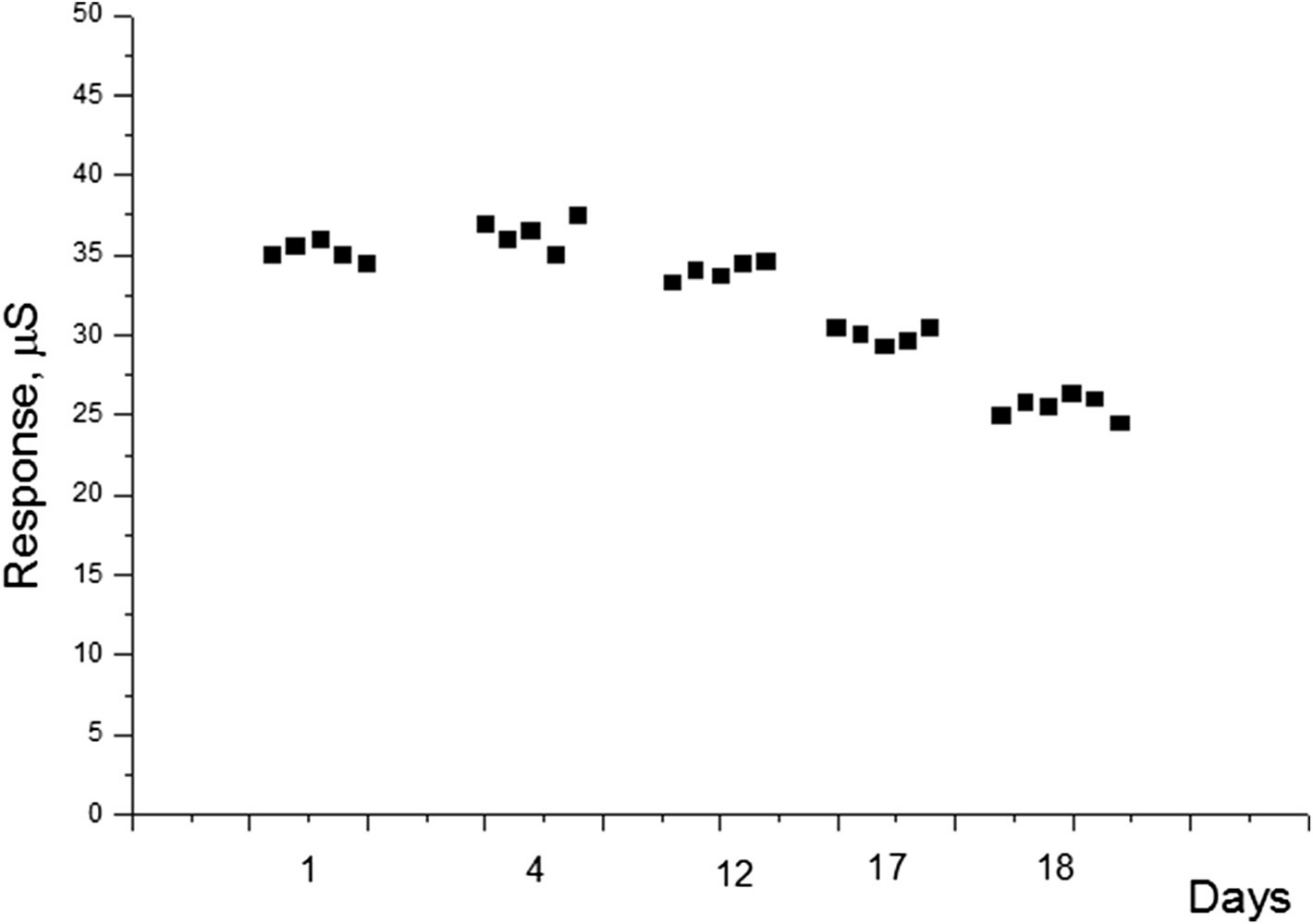
Storage stability of GOx-SME-GA biosensor. Measurements were carried out in 5 mM phosphate buffer, pH 6.5; glucose concentration was 0.2 mM

To investigate storage stability of the developed GOx-SME-GA biosensor, the responses to 0.2 mM glucose were measured during 18 days with some intervals. Between measurements, the biosensors were stored dry at 4–8 °C. The results are presented in Fig. 5. During 18 days, the responses decreased to 71 % of the initial value. Thus, this method of immobilization gives the opportunity of long-term use of biosensor.

In general, the method of immobilization with GA and silicalite provided improvement of working characteristics of the biosensors based on stainless steel transducers in comparison with other methods. Possibly, this effect can be explained by the increasing of enzyme adsorption on the electrode.

### Real Sample Analysis

The GOx-SME-GA biosensor was used for quantification of glucose in series of real samples of fruit juices and nectars. The samples were analyzed using the conductometric measurement system (see the “Measurement procedure” section). Aliquots of juices or nectars were added to the electrochemical cell, and conductometric responses were obtained. The glucose concentration was found using a standard calibration curve of the biosensor.

**Fig. 6:**
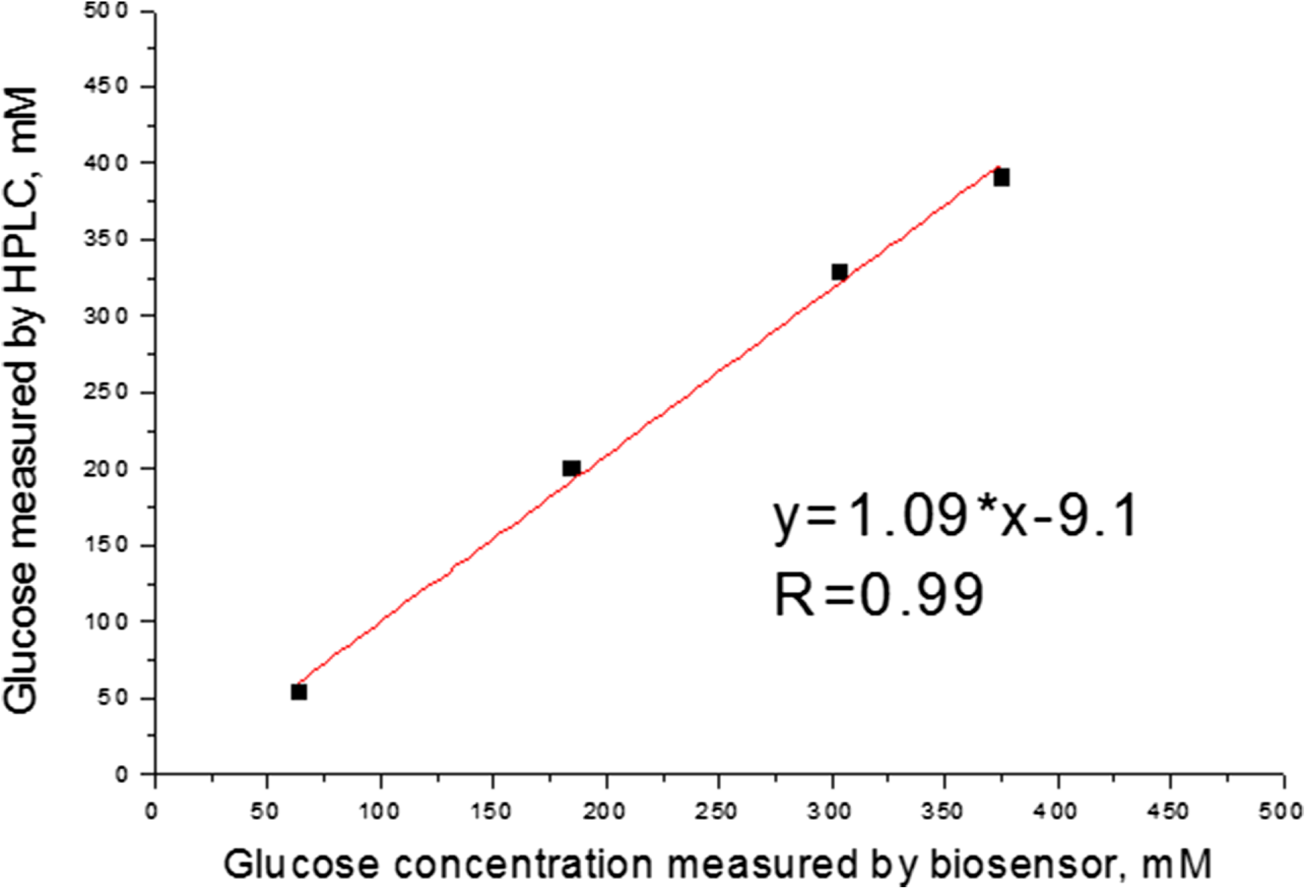
Correlation graph for biosensor and HPLC

HPLC has been chosen as a reference method due to its high sensitivity, selectivity, and accuracy. The results obtained by both methods were in good correlation (*R* = 0.99) as shown in Fig. 6 and Table 1. This encourages us for further studies aimed at commercialization, for example, optimization of conductometric GOx-SME-GA biosensor for determination of glucose in human fluids. This can help to diagnose multiple metabolic disorders with high efficiency or prevent such in case of blood glucose monitoring at home. Since GOx-SME-GA biosensor showed high correlation with HPLC and proved to be a quite stable during storage, it can be considered as a convenient platform for the creation of analytical devices for accurate, rapid, and inexpensive medical diagnostic.

**Table 1:** Comparison of the biosensor and HPLC methods for real sample analysis

Sample number/name	Glucose concentration obtained by mM (*n* = 3)	
	Biosensor	HPLC
1/Orange nectar	303.5 ± 5.3	329.9 ± 7.8
2/Orange nectar	375 ± 3.9	391.4 ± 5.3
3/Orange juice	64 ± 1	54.2 ± 0.5
4/Apple juice	185 ± 4.8	200.8 ± 6.5

## Conclusions

The new method of enzyme immobilization with GA using the silicalite-modified stainless steel electrodes (GOx-SME-GA) was compared with the traditional method of enzyme immobilization by cross-linking with GA, in which non-modified stainless steel electrodes were used (GOx-GA). The GOx-SME-GA biosensors were characterized by 4–5 times higher sensitivity than GOx-GA. The GOx-SME-GA biosensors demonstrated storage stability with only 29 % loss of activity after 18 days, satisfactory reproducibility of biosensor preparation (RSD—18 %), and good signal reproducibility (RSD of glucose determination—7 %). It was concluded that GA in complex with silicalite sufficiently enhances enzyme adsorption on stainless steel electrodes. Thus, the method of enzyme immobilization with GA along with use of silicalite is highly effective for the creation of sensitive biosensor with good signal reproducibility. The method described was used for glucose quantification in real samples of fruit juices and nectars. High correlation of this method with traditional HPLC method (*R* = 0.99) proves the opportunity of its further application and commercialization.

## Abbreviations

GA: glutaraldehyde
GOx: glucose oxidase
HPLC: high-performance liquid chromatography
ISFET: ion-selective field-effect transistor
RSD: relative standard deviation
SEM: scanning electron microscope
SME: silicalite-modified electrode
TEOS: tetraethyl orthosilicate
TPAOH: tetrapropylammonium hydroxide
